# Studies on the effect of curcumin and quercetin in the liver of male albino rats exposed to gamma irradiation

**DOI:** 10.1007/s00418-024-02300-1

**Published:** 2024-06-24

**Authors:** Amr M. Abd El-Hady, Rady M. Azzoz, Saeed M. Soliman, Ibrahim Y. Abdelrahman, Wafaa M. Khalil, Said A. Ali

**Affiliations:** 1 Radiology and Medical Imaging Technology Department, Faculty of Applied Health Sciences Technology, Misr University for Science and Technology (MUST), Cairo, Egypt; 2 Radiation Biology Department, National Centre for Radiation Research and Technology, P.O. Box 29, Nasr City, Egypt; 3 Biophysics Department, Faculty of Science, Cairo University, Giza, Egypt

**Keywords:** Curcumin, Quercetin, Liver, γ-Radiation

## Abstract

Ionizing radiation produces deleterious effects on living organisms. The present investigation has been carried out to study the prophylactic as well as the therapeutic effects of treated rats with quercetin (Quer) and curcumin (Cur), which are two medicinal herbs known for their antioxidant activities against damages induced by whole-body fractionated gamma irradiation. Exposure of rats to whole-body gamma irradiation induced a significant decrease in erythrocyte (RBC), leukocyte (WBCs), platelet count (Plt), hemoglobin concentration (Hb), hematocrit (Hct %), mean erythrocyte hemoglobin (MCH), mean corpuscular hemoglobin concentration (MCHC), and mean erythrocyte volume (MCV); a high increase in plasma thiobarbituric acid reactive substances (TBARS); a nonsignificant statistical decrease in the mean value of serum glutathione (GSH); a significant increase in plasma alanine transferase (ALT), aspartate transferase (AST), alkaline phosphates (ALP), serum total protein, serum total cholesterol levels, total triglycerides levels, high-density lipoprotein (HDL), and low-density lipoprotein (LDL) levels; and with marked histological changes and structural changes measured by Fourier transform infrared (FTIR). Applying both quercetin and curcumin pre- and postexposure to gamma radiation revealed a remarkable improvement in all the studied parameters. The cellular damage by gamma radiation is greatly mitigated by the coadministration of curcumin and quercetin before radiation exposure.

## Introduction

Exposure to ionizing radiation (IR) is inevitable since over 80% of the total average exposure comes from natural sources. Therefore, there is a pressing need to protect humans against the effects of ionizing radiation. The liver, as a very important detoxification organ in the body, is vulnerable to the deleterious effects of IR. Attempts to protect against the harmful effects of ionizing radiation by pharmacological intervention were made as early as 1949. Living organisms are always exposed to oxidative stress and toxic hazards from radiation, pollution, toxins, and others. The natural and artificial sources of ionizing radiation produce direct and indirect damage to cells. Increased reactive oxygen species (ROS) generated from the stress condition of IR cause lipid, DNA, and protein damage (Reisz et al. 2014; Abdelrahman et al. 2015).

Ionizing radiation is known to generate ROS in irradiated tissue. Because most tissues contain 80% water, the majority of radiation damage is due to aqueous free radicals, generated by the action of radiation on water. Hydroxyl radicals (•OH) are considered the most damaging of all free radicals generated in organisms (Azzam et al. 2012). The hydroxyl radical, produced during oxidative stress or radiation injury, induces a breakage in the DNA single strand (Bobrowski 2005), and DNA damage induces cell death (Carante et al. 2015). Consequently, several processes for DNA repair are activated to counteract the DNA strand breaks caused by oxidative stress. Ionizing radiation induces different types of DNA damage in bone marrow cells that may remain unrepaired. In this situation, unrepaired DNA damage may lead to cell death or genomic instability (Bagheri et al. 2018). Therefore, the risk of death or hematopoietic malignancies threatens the exposed people.

Curcumin is a major yellow pigment in turmeric ground rhizome of *Curcuma longa* Linn, which is used widely as a spice and coloring agent in several foods such as curry, mustard, and potato chips, as well as cosmetics and drugs (Okada et al. 2001; Meabed et al. 2023). The antioxidant activity of curcumin arises mainly from the scavenging of several biologically relevant free radicals that are produced during physiological processes (Lobo et al. 2010; Barzegar 2012). Quercetin, a unique bioflavonoid, is found in fruits, vegetables, grains, bark roots, stems, flowers, tea, and others (Shah et al. 2016). It is considered a powerful antioxidant flavonoid against reactive oxygen species, produced during normal oxygen metabolism or induced by exogenous damage; in addition, it possesses anti-inflammatory (Kleemann et al. 2011), vasodilatory (Perez et al. 2014), and angiogenic effects (Said et al. 2005; Zhou et al. 2015). The current study was carried out to assess the synergistic effect of both curcumin and quercetin on gamma radiation-induced disorders in rats.

## Material and methods

### Ethics approval and consent to participate

The study protocol was reviewed and approved by the Institutional Review Board of the National Center for Radiation Research and Technology, Research Ethics Committee, REC-NCRRT (approval no. 45 A/21), Chair of the committee, Prof. Dr. Mahmoud M. Ahmed. All animals were treated in accordance with ARRIVE guidelines and regulations. All methods were performed in accordance with the relevant guidelines and regulations.

### Experimental animals

A total of 42 male Sprague Dawley albino rats of about 120–150 g body weight were used in this study. They were purchased from the animal breeding house of the National Center for Radiation Research and Technology (NCRRT), Nasr City, Cairo, Egypt. Animals were housed in standard metal cages and maintained in conditions of good ventilation, normal temperatures, and humidity ranges, and kept under observation for 1 week before experimentation. Rats were fed on standard pellets containing all nutritive elements. Drinking water and food were provided ad libitum throughout the study.

### Radiation facility

Irradiation was conducted at the National Center for Radiation Research and Technology (NCRRT), Nasr City, Cairo, Egypt. A Gamma Cell-40 (Cesium 137) was employed, and the dose rate was 0.61 Gy/min during the experimental periods. Rats were whole-body exposed to 8 Gy delivered as a fractionated dose (2 Gy every 3 days).

### Preparation of quercetin and curcumin

Quercetin (Sigma-Aldrich Chemical Co., St. Louis, MO, USA) dissolved in 1 ml of normal saline, administered orally by gavage after fasting overnight at doses (1.25 g/kg) body weight (Goliomytis et al. 2014), and curcumin (Sigma-Aldrich Chemical Co., St. Louis, MO, USA) was administered orally by gavage after fasting overnight at doses (100 mg/kg) body weight (Abdel-Magied and Elkady 2019).

### Experimental design

In the present study, male albino rats weighing 120–150 g were divided into six groups of seven rats each. Group I was treated with saline and served as the control for 51 days. Group II received an oral daily dose of quercetin (1.25 g/kg) for the successive 28 days and hung up for 9 days then continued administration again for 21 days. Group III received an oral daily dose of curcumin (100 mg/kg) at the same interval time as Group II. Groups IV, V, and VI were exposed to a whole body of γ-irradiation started on day 28 of the experiment at 2 Gy/72 h (four times up to 8 Gy). Group V received a combination treatment of quercetin and curcumin (1.25 g/kg and 100 mg/kg, respectively) at the same interval time as Group II. Group VI did not receive any treatment till the end of the irradiation protocol (2 Gy/72 h, four times up to 8 Gy) and was treated postirradiation with the same dose of quercetin and curcumin for 21 days. Seven rats from each group were sacrificed after 51 days of the total time of the experiment, as presented in Table 1.

**Table 1:** The experimental design and different treatments for the groups under study

Groups	Days 1–28	Days 28–37				Days 37–51 (14 days postirradiation)
		Day 28	Day 31	Day 34	Day 35	
Group I	Saline 0.2 ml orally	Hanged up without any treatment for 9 days				Saline 0.2 ml orally
Group II	Quercetin 1.25 g/kg/day					Quercetin 1.25 g/kg/day
Group III	Curcumin 100 mg/kg/day					Curcumin 100 mg/kg/day
Group IV		2 Gy	2 Gy	2 Gy	2 Gy	
Group V	Quercetin and curcumin	2 Gy	2 Gy	2 Gy	2 Gy	Quercetin and curcumin
Group VI		2 Gy	2 Gy	2 Gy	2 Gy	Quercetin and curcumin

### Preparation of samples

At the end of the study, the animals were sacrificed after anesthesia using thiopental (Abdel-Sattar et al. 2018). Blood samples were withdrawn from each rat by heart puncture using the sterilized syringe. The blood was placed on ethylenediaminetetraacetic acid (EDTA) from Sigma-Aldrich Chemical Co., St. Louis, MO, USA, into tubes for hematological analysis and part of the samples was collected into heparin-treated tubes. Plasma samples were obtained by centrifugation at 3000 rpm for 10 min.

### Hematology analysis

#### Assessment of hematology profile

All hematological parameters: Hematological indicators including erythrocyte (RBC), leukocyte (WBC), platelet count (Plt), hemoglobin concentration (Hb), hematocrit (Hct %), mean erythrocyte hemoglobin (MCH), mean corpuscular hemoglobin concentration (MCHC), and mean erythrocyte volume (MCV) were measured by using an automated hematology analyzer model MEK-6420K, Nihon Kohden Company. Shinjuku City, Tokyo, Japan (Dacie 2017).

### Biochemical parameters

The extent of lipid peroxidation was assayed by the measurement of thiobarbituric acid reactive substances (TBARS) according to Yoshioka et al. (1979). Blood-reduced glutathione (GSH) content was determined according to the method of Beutler et al. (1963). Aspartate transferase (AST) and alanine transferase (ALT) activity were determined using the method described by Haris and Severcan (1999). Serum alkaline phosphatase (ALP) activity was measured according to Belfield and Goldberg (1971). The lipid profile tests including triglycerides, total cholesterol, high-density lipoprotein-cholesterol content (HDL-c), and low-density lipoprotein (LDL) content were evaluated using the method described by Burstein et al. (1970), Fossati and Prencipe (1982), and Moshides (1987) using a UV/VIS T60 UV/VIS spectrophotometer, PG Instruments Limited Woodway lane, Alma park, Leicestershire, UK.

### Histological methods

Small liver samples were fixed in 10% neutral buffered formalin for 48 h, embedded in paraffin after dehydration, cut into 5-μm sections and stained with hematoxylin and eosin (HE) (Burchette 2009) for the assessment of histopathological changes.

### Statistical analysis

The Statistical Package for the Social Sciences (SPSS/PC) computer program was used for statistical analysis of the results. Data were analyzed using one-way analysis of variance (ANOVA). The data were expressed as mean ± standard deviation (SD). Differences were considered significant at *P* ≤ 0.05.

### FTIR spectroscopy

Fourier transform infrared spectra of liver samples was detected on a Jasco FT/IR 460 Plus (Japan) spectrometer. KBr sandwiches were pelleted perfectly and all samples were prepared by mixing with them separately. All samples were recorded in the middle infrared frequency range in the spectral range from 400 to 4000 cm^−1^ with a speed of 2 mm/s at a resolution of 4 cm^−1^ at room temperature. The bandwidth was measured at 50% of the height of each peak. All procedures were done at the microanalytical center, Cairo University, Egypt.

## Results

### Hematological results

**Fig. 1:**
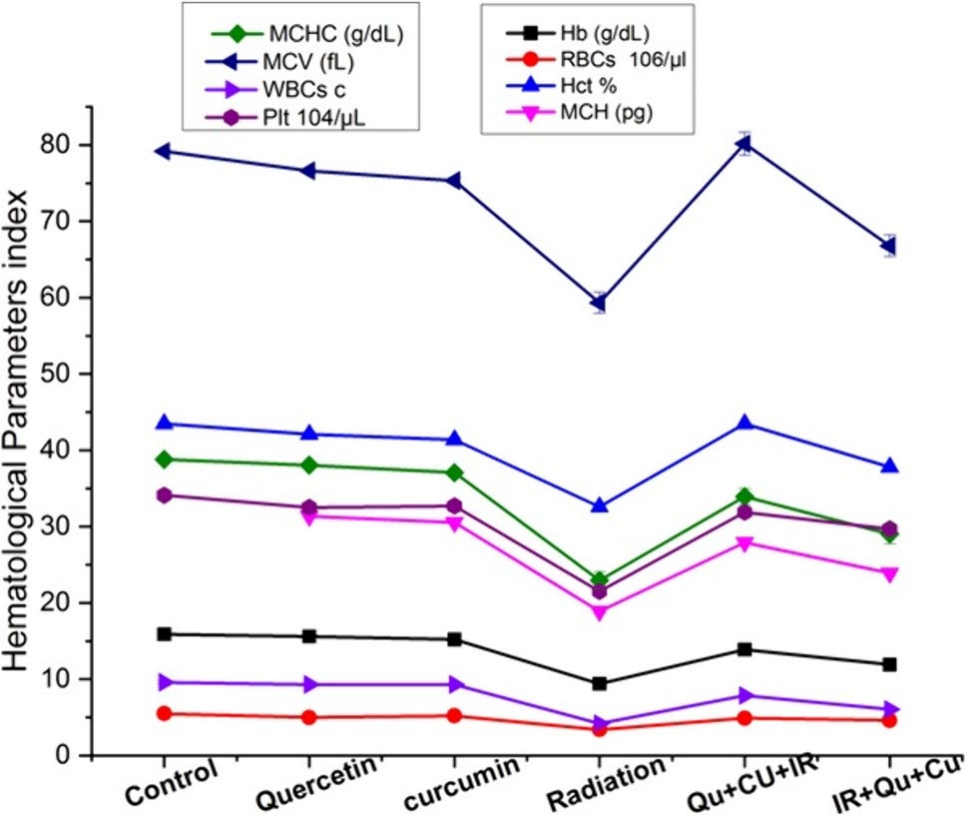
A graphical representation of the hematological parameters obtained from the different rat groups

The results of Hb, RBCs, Hct %, MCH, MCHC, MCV, WBCs, and platelet count (Plt) showed no statistical difference in their mean values following oral administration of quercetin or curcumin for 51 days as compared with the control group. Exposure of rats to whole-body gamma irradiation at fractionated doses (2 Gy, four times, every 3 days) up to 8 Gy triggered a highly significant statistical decrease in Hb, RBCs, MCH, MCHC, WBC count, and platelet count (*P* < 0.01) with a significant statistical decrease in the Hct %, MCV on the 14th day following irradiation process as compared to the control values (*P* < 0.05). The dual oral administration of both quercetin and curcumin pre-irradiation induced a highly significant increase in all studied parameters (*P* < 0.01) throughout the experimental times as compared with the corresponding irradiated group value. The co-administration of both quercetin and curcumin postirradiation showed a highly significant increase in the RBC count, WBCs, and platelet count on the 14th day (*P* < 0.01) as compared with the irradiated group values, indicating that administration of both quercetin and curcumin before exposure to gamma radiation was more effective than their postirradiation administration. All the hematological parameters are listed in Table 2 and illustrated in Fig. 1.

**Table 2:** Hematological parameters Hb, RBCs, Hct %, MCH, MCHC, MCV, WBCs, and Plt for the six rat groups

Animal group	Hb (g/dL)	RBCs 10^6^/µl	Hct %	MCH (pg) Hb/RBC	MCHC (g/dL) Hb/Hct	MCV (fL) Hct/RBC	WBCs c	Plt 10^3^/µL
Control	15.9 ± 0.29	5.5 ± 0.29	43.5 ± 0.19	31.95 ± 0.14	38.79 ± 0.19	79.17 ± 0.19	9.59 ± 0.19	341 ± 1.19
Quercetin	15.6 ± 0.23	5.0 ± 0.30	42.1 ± 0.33	31.35 ± 0.13	38.06 ± 0.33	76.62 ± 0.33	9.30 ± 0.17	325 ± 1.33
Change from control (%)	−1.88%	−9.09%	−3.21%	−1.87%	−1.88%	−3.22%	−3.02%	−4.69%
*P*-value versus control	(*P* > 0.05)	(*P* > 0.05)	(*P* > 0.05)	(*P* > 0.05)	(*P* > 0.05)	(*P* > 0.05)	(*P* > 0.05)	(*P* > 0.05)
Curcumin	15.2 ± 0.24	5.2 ± 0.45	41.4 ± 0.24	30.55 ± 0.22	37.08 ± 0.42	75.34 ± 0.42	9.32 ± 0.18	327 ± 1.42
Change from control (%)	−4.40%	−5.45%	−4.82%	−4.38%	−4.40%	−4.83%	−2.81%	−3.83%
*P*-value versus control	(*P* > 0.05)	(*P* > 0.05)	(*P* > 0.05)	(*P* > 0.05)	(*P* > 0.05)	(*P* > 0.05)	(*P* > 0.05)	(*P* > 0.05)
Radiation	9.4 ± 0.16	3.4 ± 0.09	32.6 ± 0.13	18.89 ± 0.26	22.93 ± 1.1	*59.33* ± *1.4*	4.17 ± 0.21	215 ± 1.5
Change from control (%)	−40.88%	−38.18%	−25.05%	−40.87%	−40.88%	*−25.05%*	−56.51%	−36.95%
*P*-value versus control	(*P* < 0.01)	(*P* < 0.01)	(*P* < 0.05)	(*P* < 0.01)	(*P* < 0.01)	*(P* < *0.05)*	(*P* < 0.01)	(*P* < 0.01)
Quercetin + curcumin + radiation	13.9 ± 0.5	4.9 ± 0.22	43.5 ± 0.14	27.93 ± 0.34	33.91 ± 1.08	80.17 ± 1.5	7.86 ± 0.13	319 ± 2.2
Change from radiation (%)	47.87%	44.11%	33.43%	47.85%	47.88%	35.12%	88.48%	48.37%
*P*-value versus radiation	(*P* < 0.01)	(*P* < 0.01)	(*P* < 0.01)	(*P* < 0.01)	(*P* < 0.01)	(*P* < 0.01)	(*P* < 0.01)	(*P* < 0.01)
Radiation + quercetin + curcumin	11.9 ± 0.04	4.6 ± 0.13	37.8 ± 0.22	23.91 ± 0.38	29.03 ± 1.3	66.79 ± 1.4	6.05 ± 0.10	297 ± 1.64
Change from radiation (%)	26.59%	35.29%	15.95%	26.57%	26.60%	12.57%	45.08%	38.13%
*P*-value versus radiation	(*P* < 0.05)	(*P* < 0.01)	(*P* > 0.05)	(*P* < 0.05)	(*P* < 0.05)	(*P* > 0.05)	(*P* < 0.01)	(*P* < 0.01)

### Biochemical results

#### Plasma thiobarbituric acid reactive substances (TBARS) concentration

**Fig. 2:**
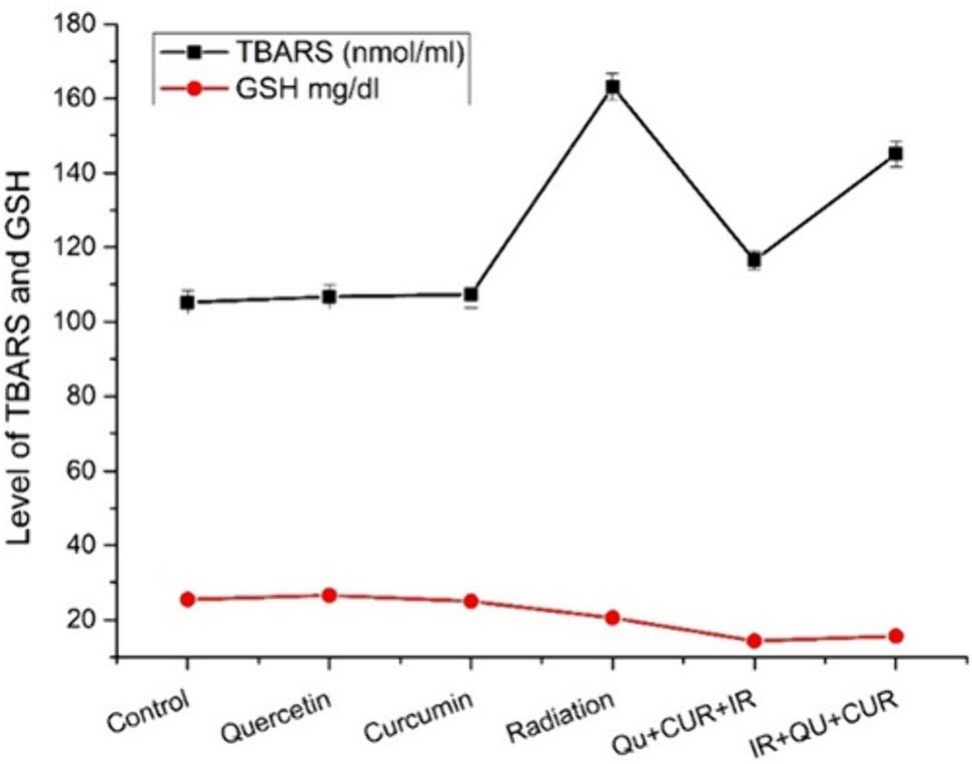
A graphical representation of the TBARS and GSH obtained of the different rat groups

The oral administration of Quer or Cur for consecutive 51 days showed no significant differences in plasma TBARS concentration as compared with the control untreated group. Exposure of rats to the fractionated doses of $\gamma$-irradiation at up to 8 Gy resulted in highly significant increases in the TBARS concentration (*P* < 0.01) as compared with the control values. Administration of both Quer and Cur before irradiation showed a significant decrease in the TBARS concentration (*P* < 0.05) as compared with the corresponding irradiated group, while their postirradiation administration showed a nonsignificant decrease (*P* > 0.05) as compared with the corresponding irradiated group, the recorded values are shown graphically in Fig. 2 and listed in Table 3.

**Table 3:** Biochemical parameters, TBARS and GSH recorded for the different rat groups

Animal group	TBARS (nmol/ml)	GSH mg/dl
Control	105.24 ± 3.19	25.42 ± 1.13
Quercetin Changes from control (%) *P*-value versus control	106.69 ± 3.40 1.37% (*P* > 0.05)	26.59 ± 1.28 4.60% (*P* > 0.05)
Curcumin Changes from control (%) *P*-value versus control	107.30 ± 3.47 1.95% (*P* > 0.05)	24.97 ± 1.04 −1.77% (*P* > 0.05)
Radiation Changes from control (%) *P*-value versus control	163.10 ± 3.61 54.97% (*P* < 0.01)	20.51 ± 0.95 −19.31% (*P* > 0.05)
Quercetin + curcumin + radiation Change from radiation (%) *P*-value versus radiation	116.5 ± 2.57 −28.57% (*P* < 0.05)	14.28 ± 0.78 −30.37% (*P* < 0.01)
Radiation + quercetin + curcumin Change from radiation (%) *P*-value versus radiation	145.03 ± 3.38 −11.07% (*P* > 0.05)	15.57 ± 0.88 −24.08% (*P* < 0.05)

### Blood glutathione (GSH) content

The oral administration of Quer or Cur for consecutive 51 days showed no significant differences in blood GSH content as compared with the control untreated group. Exposure of rats to the fractionated doses of $\gamma$-irradiation at up to 8 Gy resulted in a nonsignificant statistical decrease in the mean value of serum GSH level (*P* > 0.05) as compared with the control group. Administration of both Quer and Cur before irradiation induced a highly significant decrease in the GSH level (*P* < 0.01), while their postirradiation administration showed a significant statistical decrease in its level (*P* < 0.05) as compared with the corresponding irradiated group. The recorded values are displayed in Fig. 2 and Table 3.

### Liver function results

**Fig. 3:**
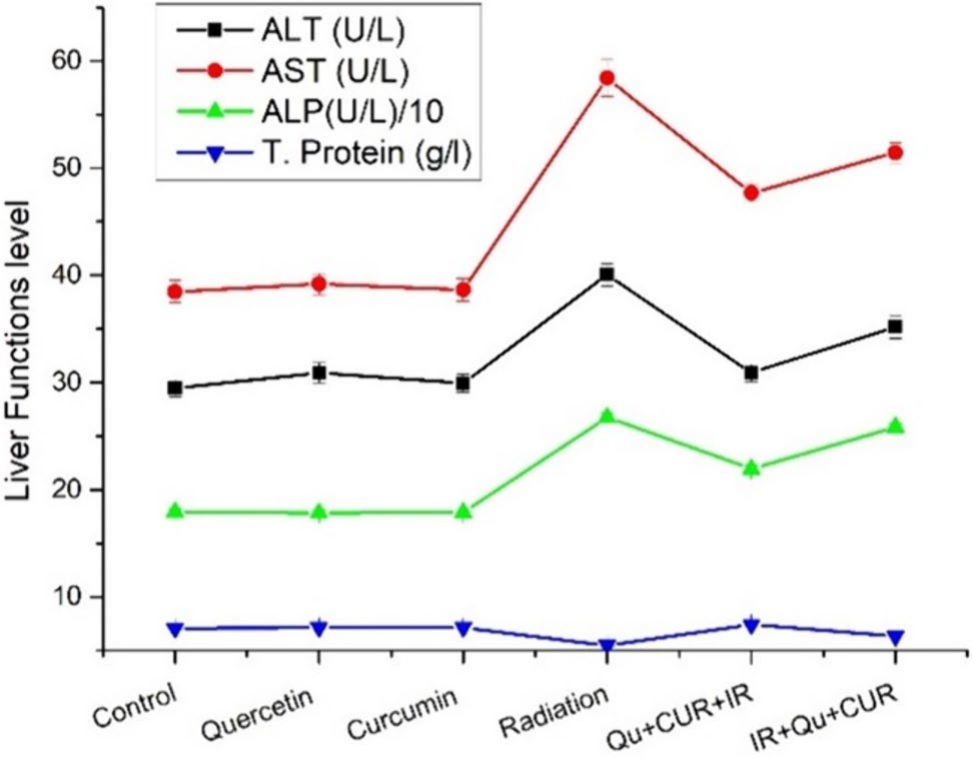
A graphical representation of the liver function parameters obtained from the different rat groups

As presented in Table 4, and depicted in Fig. 3, oral supplementation of Quer or Cur for consecutive 51 days did not cause a significant statistical difference in the serum ALT, AST, ALP activities, or serum total protein content as compared with the control group values. A significant statistical increase was noticed in ALT, AST, and ALP activities on the 14th day (*P* < 0.01) in the $\upgamma$-irradiated rat group with a significant decrease in the serum total protein values (*P* < 0.05) as compared with the control group values. Pre-irradiation administration of both Quer and Cur resulted in a significant decrease in serum ALT activity (*P* < 0.05) with a highly significant statistical increase (*P* < 0.01) in the total protein content, otherwise, there was a nonsignificant decrease in both AST and ALP activities (*P* > 0.05) as compared with the irradiated group recorded values. Oral administration of both Quer and Cur postirradiation resulted in a nonsignificant amelioration of the radiation-induced decrease of ALT, AST, and ALP activities values (*P* > 0.05) with an insignificant increase in the total protein content (*P* > 0.05) as compared with the corresponding irradiated group values.

**Table 4:** Liver function parameters recorded for the different rat groups

Animal groups	ALT (U/L)	AST (U/L)	ALP (U/L)*10	Total protein (g/l)
Control	29.46 ± 0.81	38.47 ± 1.05	17.918 ± 0.307	7.06 ± 0.11
Quercetin	30.9 ± 0.92	39.18 ± 0.97	17.827 ± 0.299	7.12 ± 0.09
Changes from control (%)	4.88%	1.84%	−0.50%	0.84%
*P*-value versus control	(*P* > 0.05)	(*P* > 0.05)	(*P* > 0.05)	(*P* > 0.05)
Curcumin	29.94 ± 0.84	38.64 ± 1.04	17.868 ± 0.281	7.13 ± 0.10
Changes from control (%)	1.62%	0.44%	−0.27%	0.99%
*P*-value versus control	(*P* > 0.05)	(*P* > 0.05)	(*P* > 0.05)	(*P* > 0.05)
Radiation	40.06 ± 1.04	58.39 ± 1.71	26.760 ± 0.352	5.48 ± 0.052
Changes from control (%)	39.37%	51.78%	49.34%	−22.37%
*P*-value versus control	(*P* < 0.01)	(*P* < 0.01)	(*P* < 0.01)	(*P* < 0.05)
Quercetin + curcumin + radiation	30.89 ± 0.77	47.68 ± 0.83	21.94 ± 0.337	7.41 ± 0.24
Change from radiation (%)	−22.89%	−18.34%	−18.01%	35.21%
*P*-value versus radiation	(*P* < 0.05)	(*P* > 0.05)	(*P* > 0.05)	(*P* < 0.01)
Radiation + quercetin + curcumin	35.17 ± 1.06	51.43 ± 0.96	25.831 ± 0.345	6.33 ± 0.13
Change from radiation (%)	−12.20%	−11.91%	−3.47%	15.51%
*P*-value versus radiation	(*P* > 0.05)	(*P* > 0.05)	(*P* > 0.05)	(*P* > 0.05)

### Lipid profile results

**Fig. 4:**
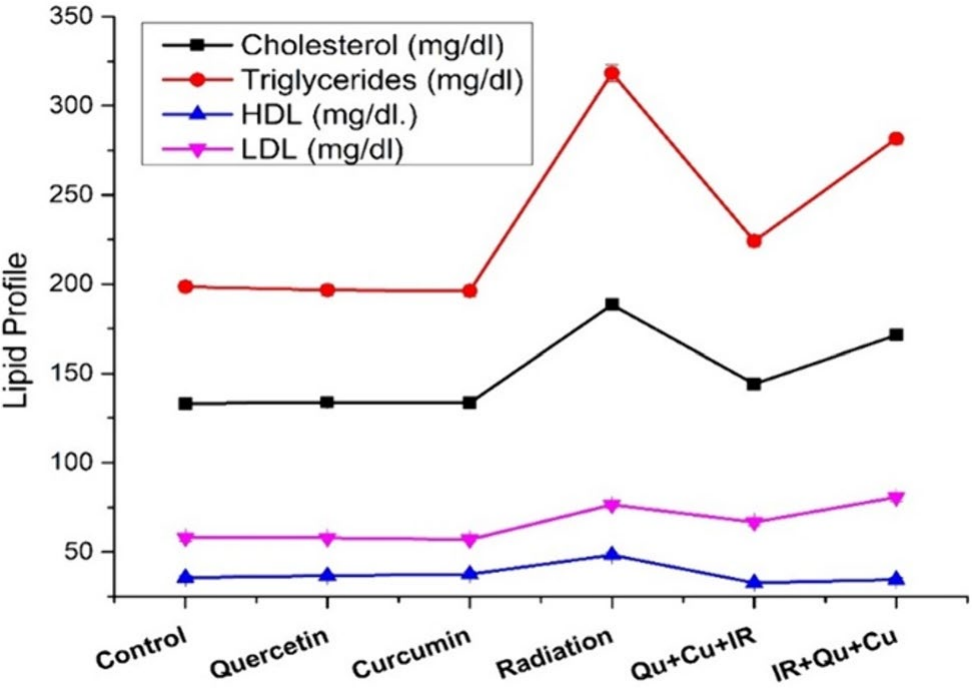
A graphical representation of the lipid profile parameters obtained from the different rat groups

No significant differences were detected in the serum total cholesterol, triglycerides, HDL, and LDL levels between the control group and the groups treated with quercetin or curcumin. The current experiment elucidated a highly significant elevation (*P* < 0.01) in the serum total cholesterol, triglycerides, HDL, and LDL concentrations following fractionated doses of γ-radiation as compared with those of the control rats. Pre-irradiation treatment of rats with both Quer and Cur induced a significant decrease in the cholesterol level (*P* < 0.05) with a highly significant decrease in both triglyceride and HDL levels (*P* < 0.01) and a nonsignificant decrease in the LDL level (*P* > 0.05) as compared with the irradiated group values, whereas, its postirradiation treatment exerted a nonsignificant decrease in the serum total cholesterol, triglycerides, and LDL (*P* > 0.05) levels with a significant decrease in the HDL level (*P* < 0.05) as compared with the irradiated group values (Table 5). A graphical illustration of the different lipid parameters recorded is shown in Fig. 4.

**Table 5:** Lipid profile parameters recorded for the different rat groups

Animal group	Cholesterol (mg/dl)	Triglycerides (mg/dl)	HDL (mg/dl.)	LDL (mg/dl)
Control	133.26 ± 2.27	198.47 ± 3.54	35.61 ± 1.14	57.96 ± 2.14
Quercetin	133.86 ± 2.31	196.65 ± 3.03	36.66 ± 1.13	57.87 ± 1.70
Changes from control (%)	0.45%	−0.91%	2.94%	−0.15%
*P*-value versus control	(*P* > 0.05)	(*P* > 0.05)	(*P* > 0.05)	(*P* > 0.05)
Curcumin	133.61 ± 2.17	196.18 ± 3.14	37.47 ± 1.29	56.91 ± 1.19
% of changes from control	0.26%	−1.15%	5.22%	−1.81%
*P*-value versus control	(*P* > 0.05)	(*P* > 0.05)	(*P* > 0.05)	(*P* > 0.05)
Radiation	188.40 ± 2.29	318.32 ± 4.66	48.28 ± 1.40	76.46 ± 2.07
Changes from control (%)	41.37%	60.38%	35.57%	31.91%
*P*-value versus control	(*P* < 0.01)	(*P* < 0.01)	(*P* < 0.01)	(*P* < 0.01)
Quercetin + curcumin + radiation	144.14 ± 2.13	224.14 ± 3.25	32.64 ± 1.15	66.68 ± 2.15
Change from radiation (%)	−23.49%	−29.58%	−32.39%	−12.79%
*P*-value versus radiation	(*P* < 0.05)	(*P* < 0.05)	(*P* < 0.01)	(*P* > 0.05
Radiation + quercetin + curcumin	171.42 ± 2.18	281.55 ± 3.19	34.47 ± 1.19	80.64 ± 2.39
Change from radiation (%)	−9.01%	−11.55%	−28.60%	5.46%
*P*-value versus radiation	(*P* > 0.05)	(*P* > 0.05)	(*P* < 0.05)	(*P* > 0.05)

### Histopathological results

**Fig. 5:**
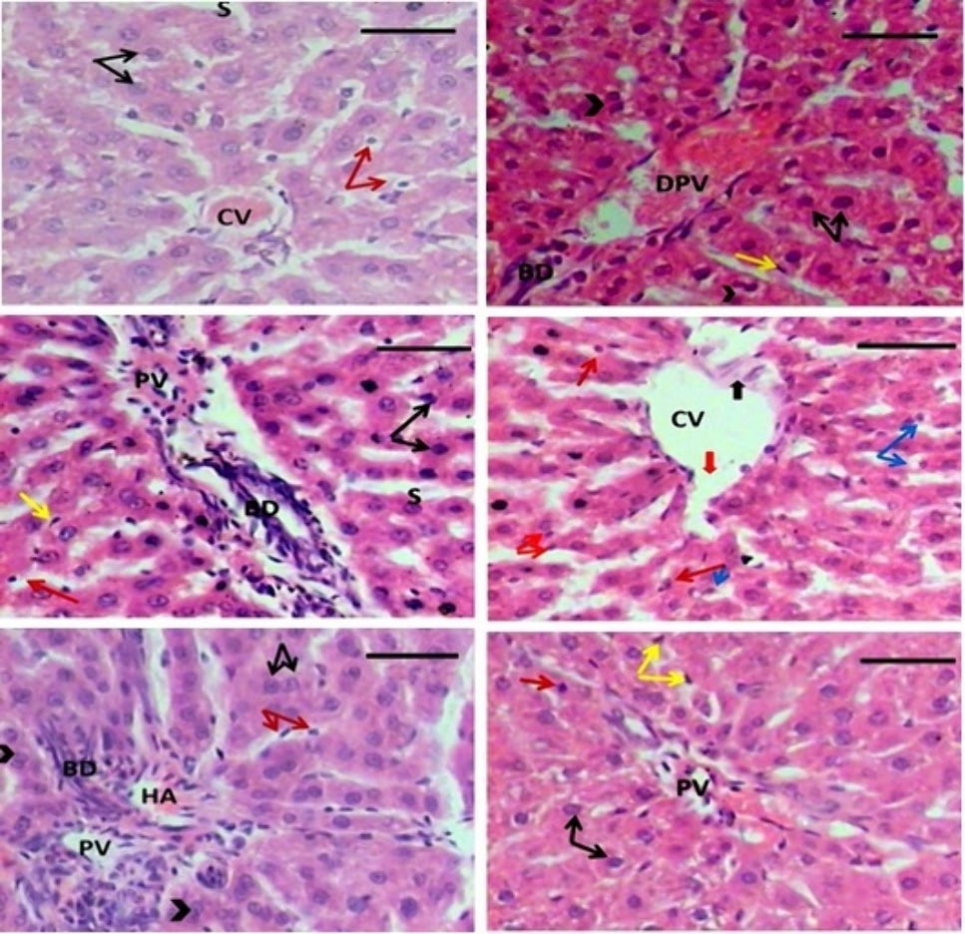
Photomicrograph from the liver of rats showing: **a** The control group showing normal central vein (CV), cords of healthy hepatocytes with central vesicular nuclei radiating from it and separated from each other by blood sinusoids (S). Few cells have pyknotic nuclei (red arrows). **b** and **c** The Quer and Cur groups showed portal vein (PV) and normal bile duct (BD), healthy hepatocytes with central vesicular nuclei (black arrows), and some cells appeared binucleated (arrowheads) with activated Kupffer cells (yellow arrows). **d** Radiation (R) group showing dilated congested central vein (CV) with discontinuation (thick red arrow) and delamination (thick black arrow) of its lining, hepatocytes with degenerative changes as some with pyknotic nuclei (red arrow) and others with vacuolated cytoplasm (blue arrows). **e** Quer + Cur + R group showing normal portal vein (PV), hepatic artery (HA), bile duct (BD), and hepatocytes (black arrows) some with binucleated cells (arrowheads). **f** R + Quer + Cur group showing normal portal vein (PV), sinusoids, hepatocytes, some with prominent nucleolus (black arrows), and others with pyknotic nuclei (red arrow) and activated Kupffer cells (yellow arrows). Scale bar: 30 µm

Histological examination of a liver section of the control group revealed a normal histological appearance of the hepatocytes, which are polygonal in shape and radially disposed of in the liver lobule. Each hepatic cell has a centrally located nucleus with one or two prominent nuclei. Occasionally, the liver cells appear binucleated. The spaces between the hepatic plates contain the liver sinusoids with phagocytic cells of the mononuclear phagocyte series known as Kupffer cells. Each hepatic lobule has a central vein at its core (Fig. 5a). Liver sections of rats of the quercetin group showed the same normal histological appearance including a normal central vein, dilated portal vein, and bile duct. Hepatocytes appeared with central vesicular nuclei where some hepatocytes are double nucleated as a sign of regeneration. Kupffer cells appeared activated as seen in Fig. 5b. Curcumin-treated rats showed the same normal architecture of hepatic parenchymal cells with the blood sinusoids that appeared occupied by blood cells with activated Kupffer cells. The portal tract appeared normally formed of the portal vein and bile duct (Fig. 5c**)**.

Whole-body exposure of rats of the current experiment to 8 Gy gamma irradiation delivered as a fractionated dose (2 Gy every 3 days) showed loss of the normal hepatic architectures with dilated central vein with corrugated walls and widened blood sinusoids. Some hepatocytes appeared degenerated with pyknotic nuclei and vacuolated cytoplasm (Fig. 5d).

Administration of both quercetin and curcumin before gamma radiation exposure showed more or less normal hepatic architecture with the normal portal vein and bile duct. Hepatocytes appeared healthy with central vesicular nuclei, some of which appeared binucleated as a sign of regeneration as depicted in (Fig. 5e). Administration of both quercetin and curcumin following gamma radiation exposure showed signs of recovery and tissue repair indicated by the well-developed hepatic architecture with a normal portal tract formed of the portal vein and bile duct, and widened blood sinusoids were still detected. Most of the hepatocytes appeared with central vesicular nuclei, while others have pyknotic nuclei (Fig. 5f).

### FTIR spectroscopy

**Fig. 6:**
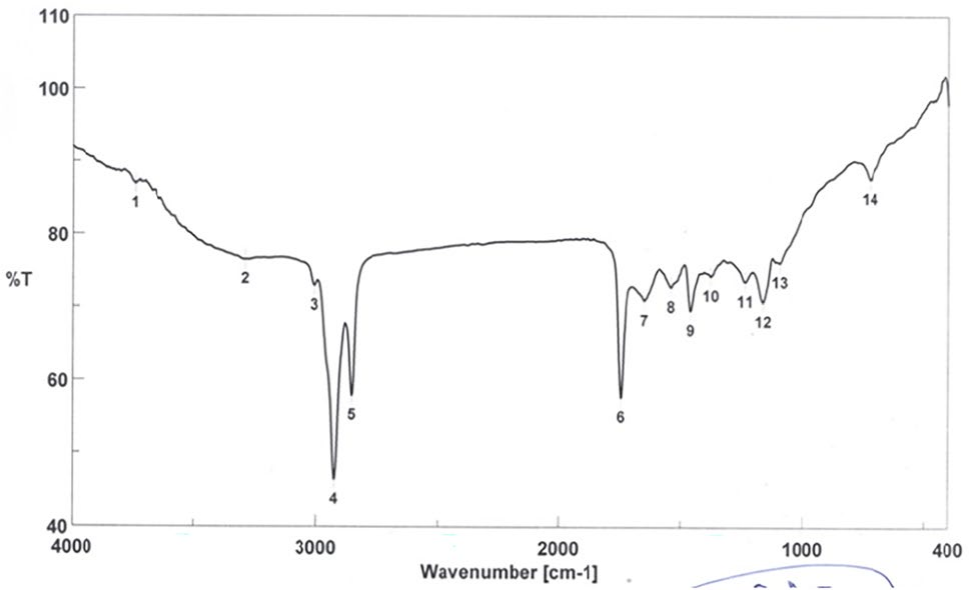
The average FTIR spectra of control liver tissues in the 4000–400 cm^−1^ region

The average FTIR spectra of control liver tissues in 4000–400 cm^−1^ regions is shown in Fig. 6. The main bands are labeled in the figure, and the band assignments are given in Table 6, (Stuart 1997; Haris and Severcan 1999; Movasaghi et al. 2008; Bozkurt et al. 2010; Severcan et al. 2010; Cakmak et al. 2011).

**Table 6:** Band assignments of major transmissions in IR spectra of control liver tissue in 4000–400 cm^−1^ regions

Peak no.	Wavenumber (cm^−1^)	Definition of the spectral assignment [28–33]
1	3741	OH stretching
2	3289	Amide A: mainly N–H stretching of proteins
3	3006	Olefinic=CH stretching vibration: unsaturated lipids, cholesterol esters
4	2924	CH2 antisymmetric stretch: mainly lipids
5	2854	CH2 symmetric stretch: mainly lipids
6	1745	Saturated ester C=O stretch: Phospholipids, cholesterol esters
7	1651	Amide |: Protein (80% C=O stretching, 10% N–H bending, 10% C–N stretching
8	1539	Amide II: Protein (60% N–H bending, 40% C–N stretching
9	1461	CH2 bending: mainly lipids, protein
10	1379	COO-symmetric stretch: fatty acids and amino acids
11	1237	PO2 antisymmetric stretch: nucleic acids, phospholipids
12	1164	CO–O–C antisymmetric stretching: glycogen and nucleic acids
13	1097	PO2 symmetric stretch: nucleic acids, phospholipids

**Fig. 7:**
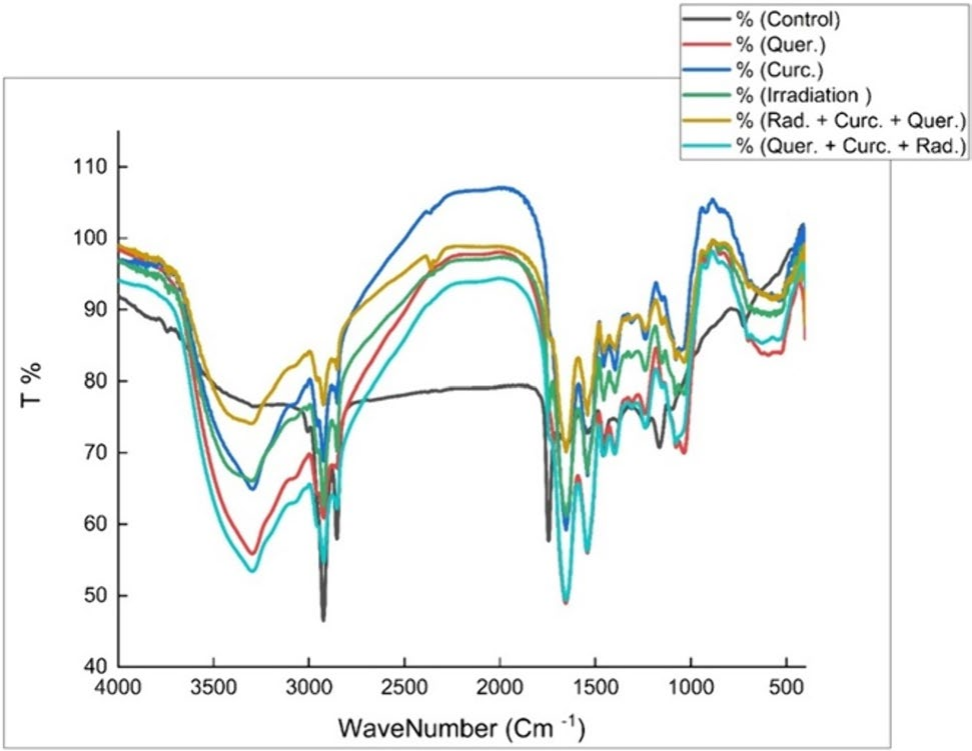
The average FTIR spectra of control, quercetin, curcumin, irradiated, and combined quercetin–curcumin before and after irradiation-treated liver tissues in 4000–400 cm^−1^ region

The average FTIR spectra of control, Quer, Cur, irradiated and combined Quer-Cur before and after irradiation-treated rat liver tissues in 4000–400 cm^−1^ region is shown in Fig. 7. The figure reveals prominent differences between the average spectra belonging to the different groups. Subtle changes in a band shape, band position, and band intensity of vibrational bands represent changes in biomolecule concentration, composition, and structure. It was observed that the broad peak of the OH group, CH_2_, C=O, amide I, and C=C, respectively, had increased in intensity by varying the dopant material. All peaks before 1600 cm^−1^ decreased in intensity by varying dopants. Peaks after 1600 cm^−1^ increased in intensity by adding quercetin and decreased by adding curcumin. By adding quercetin, the peak positions were shifted to a lower wavenumber, while by adding curcumin, there were some fluctuations (many peaks increase in wavenumber and the others decrease). The reduced wavenumber may be due to the dopant material not interacting properly with the liver’s protein. The contrary is true; the increase in wavenumber is caused by the strong interaction between proteins and dopant material through the formation of hydrogen bonds.

The radiation effect on liver tissues was indicated by the shift of 3289 cm^−1^ of NH stretching protein amide A to a higher intensity concerning the control and the disappearance of the OH stretching. It also shows a high decrease of the peaks 1745, 1651, 1539, and 1461 cm^−1^ of phospholipids, amide 1 and amide 2 and lipid–protein, respectively, to a lower intensity concerning the control. There is also a shift in the peaks of 3006 cm^−1^ for olefinic CH stretching for lipid and cholesterol, 2924 cm^−1^ CH_2_ for antisymmetric lipids, and a peak of 2854 cm^−1^ for CH_2_ symmetric lipids to lower intensity indicating the direct effect of radiation on liver tissues. It is shown from the figure that the radiation effect of the post-treated quercetin–curcumin group showed a decrease in the intensity of all peaks indicating its effect against radiation effect. There was a higher shift to higher intensity values for the irradiated pretreated quercetin–curcumin group in a close match with the control group. The combined doping of both quercetin and curcumin before and after irradiation showed a more significant effect in ameliorating the radiation effect on liver tissues, nearly restoring all the peaks to the control of the unirradiated one.

## Discussion

Due to the widespread usage of radiation in diagnosis, therapy, and industry, pharmacological intervention could be the most potent strategy to counter or ameliorate the injurious effects of radiation exposure (Singh and Seed 2020). The histological examination results included in the current study revealed that a fractionated dose of 8 Gy of γ-irradiation induced different histopathological changes, indicated by loss of the normal hepatic architecture, which began with dilatation of the central vein and hepatic blood sinusoids, vacuolation and degeneration of its cytoplasm, pyknosis, and activation of Kupffer cells. The current results run in parallel with those reported previously (Guryev 2005; El Adham et al. 2022).

Such alterations could be attributed to ionizing radiation, which produces damaging cellular effects through water radiolysis, resulting in the release of reactive oxygen species (ROS) in cells and the reduction of cellular antioxidants including GSH and enzymatic antioxidants. ROS can induce inflammatory reactions (Kumar et al. 2014). Radiation damage to living tissues was reported before to be a result of the overproduction of ROS (Abd El-Rahman and Sherif 2015). ROS overproduction increases lipid peroxidation in living cells, which increases oxidative stress enhanced by the disturbances of oxidants/antioxidants damage of the biological macromolecules including lipids, carbohydrates, proteins, and nucleic acids, resulting in disturbance of the cellular homeostasis and the production of other reactive oxygen molecules that initiate more oxidative damage (Birben et al. 2012). The results also showed that administration of both quercetin and curcumin antioxidants before and after radiation exposure decreased liver damage; however, the deleterious effects of gamma radiation were reduced more efficiently by its pre-irradiation administration, which is confirmed by restoring all the peaks of the FTIR spectrum to be matching the control unirradiated group.. Curcumin is classified as a radioprotective agent because of its capacity to reduce oxidative stress and inflammatory reactions (Jagetia 2007). By preserving the oxidative balance inside cells, quercetin exhibited strong antioxidant properties as well (Xu et al. 2019).

## Conclusions

Data from the present experiment showed that synergistic treatment of gamma-irradiated rats with both quercetin and curcumin antioxidants appears susceptible to lowering the statistical increase recorded in the ALT, AST, ALP, total cholesterol, triglycerides, HDL and LDL concentrations. The radioprotective efficiency of both antioxidants could be attributed to their role in scavenging free radicals (Chittasupho et al. 2022). Quercetin and curcumin are capable of alleviating intracellular oxidative stress (Zhao et al. 2017). Curcumin exerts various biological functions including antioxidation and anti-inflammation, and quercetin also has an important role in suppressing the expression of oxidative stress and inflammatory markers (Panchal et al. 2012). It could be concluded that quercetin and curcumin may exert a beneficial impact on gamma irradiation-induced liver injury in rats.

## Data Availability

All data are available from the corresponding author upon request.
